# Effects of Compulsory Schooling on Mortality: Evidence from Sweden 

**DOI:** 10.3390/ijerph10083596

**Published:** 2013-08-13

**Authors:** Martin Fischer, Martin Karlsson, Therese Nilsson

**Affiliations:** 1 Ruhr Graduate School in Economics (RGS Econ), University of Duisburg-Essen, Schützenbahn 70, Essen 45127, Germany; 2 Chair of Health Economics, University of Duisburg-Essen, Schützenbahn 70, Essen 45127, Germany; E-Mail: martin.karlsson@uni-due.de; 3 Department of Economics and Centre for Economic Demography (CED), Lund University, Lund 22007, Sweden; E-Mail: therese.nilsson@nek.lu.se; 4 Institute of Industrial Economics (IFN), Stockholm 10215, Sweden

**Keywords:** returns to schooling, education reform, mortality

## Abstract

Theoretically, there are several reasons to expect education to have a positive effect on health. Empirical research suggests that education can be an important health determinant. However, it has not yet been established whether education and health are indeed causally related, and the effects found in previous studies may be partially attributable to methodological weaknesses. Moreover, existing evidence on the education-health relationship generally uses information of fairly recent schooling reforms, implying that health outcomes are observed only over a limited time period. This paper examines the effect of education on mortality using information on a national roll-out of a reform leading to one extra year of compulsory schooling in Sweden. In 1936, the national government made a seventh school year compulsory; however, the implementation was decided at the school district level, and the reform was implemented over 12 years. Taking advantage of the variation in the timing of the implementation across school districts, by using county-level proportions of reformed districts, census data and administrative mortality data, we find that the extra compulsory school year reduced mortality. In fact, the mortality reduction is discernible already before the age of 30 and then grows in magnitude until the age of 55–60.

## 1. Introduction

There is a well-known empirical association between education and health. It has been observed in several countries and time periods and for a wide range of health measures. For example, the life expectancy of male American university graduates at age 25 was 56 years in 2006, compared to 47 years for individuals without a high school degree. For females, the corresponding figure was 60 for graduates and 52 for high school dropouts. Besides, there is evidence suggesting that recent improvements in life expectancy have been concentrated in higher educational groups: since 1996, university graduates have increased their life expectancy at 25 by 1–2 years, whereas no improvement has been observed for the lowest educational group [1].

In Sweden, pecuniary returns to education appear to be much smaller than in the U.S. An American university graduate can expect to earn 60–85% more than someone with secondary education only, and individuals with educational attainment below the secondary level have earnings 30–35% below those of high school graduates. In Sweden, the premium is much lower for both levels, and the college premium is as low as 13% for individuals aged 25–34 [2]; income taxes even out these smaller differences even further.

Despite these relatively small differences in disposable income between educational groups, Sweden, however, just like the U.S., exhibits a striking education gradient in life expectancies, and these differences show no tendency of abating. In the year 2000, life expectancy at 30 was 51.5 years for males with a university degree; for individuals with less than a secondary education, it was 46.6 years. Ten years later, university graduates had increased their life expectancy to 53.2 years, compared to 48.0 years for the group with the lowest educational attainment [3].

The study of financial returns to education have a long history in economics, and over the years, a consensus has emerged that an additional year in secondary education is associated with a substantial increase in wages, in particular for individuals from disadvantaged backgrounds [4]. When non-pecuniary outcomes, such as health, are concerned, there is much less evidence and, also, much less agreement between different contexts. It remains unclear to what extent the disparities in life expectancies mentioned above reflect a causal impact of education on health. An education gradient would also arise if there were reverse causation, so that childhood health—which is known to be a strong predictor of adult health—affects the number of years of schooling. Likewise, there may be a third factor affecting both education and health—such as family background, time preferences or cognitive abilities. Cutler and Lleras-Muney [5], however, conclude that there are reasons to be skeptical about these two explanations: the widening of disparities can hardly be driven by child health, which has been improving over time, and controlling for potential confounders tends to only slightly mitigate the correlation between education and health.

If education has a causal effect on health, it is important to consider potentially relevant mechanisms. Traditionally, the main attention of economists has been devoted to the role of education in facilitating the production of health: individuals with higher human capital likely combine inputs in the health production process in a more efficient manner [6]. However, alternative explanations seem equally plausible: education may also affect an individual’s working conditions, preferences, rank in society and social networks—all of which are instrumental to maintaining good health [5].

Identification problems and potential mechanisms aside, one reason for the lack of a consensus regarding the effects of education on health is probably the wide variety of health outcomes that have been considered in the literature: previous studies have considered self-reported health (SAH), hospitalizations, weight and BMI, long-term illness, biomarkers, mortality and health-related behaviors [7,8,9,10]. However, also in cases where the same exogenous variation is used to estimate the impact on the same type of outcome, researchers disagree about the effect. For example, Oreopoulos [11] and Silles [12] both use extensions of compulsory schooling in Britain to estimate the effect of schooling on SAH. Both studies claim to find substantial positive effects of the reforms on SAH. In a recent study, however, Clark and Royer [10] challenge this view and report effects that are small and insignificant—and attribute the effects found in previous studies to methodological weaknesses. In an online corrigendum, Oreopoulos later, however, presents evidence suggesting that there is no effect on SAH. This debate clearly highlights the importance of carefully scrutinizing the identification strategy.

In this paper, we use a change in compulsory schooling legislation in Sweden to estimate the effect of schooling on mortality. In 1936, the national government decide that a seventh school year should be introduced in all school districts before 1949. The reform was typically implemented earlier in urban districts than in rural districts, but there is also general variation in the timing of implementation between districts, which facilitates the identification of the effect.

The reform we consider has not been used before to estimate the effects of schooling, but there are several reasons why we think it is very suitable for analyzing the effects of education on health. First, it represents a relatively large expansion of compulsory schooling, so it can be expected that the estimated effects (if any) are relatively large. Second, whereas the next reform that followed in Sweden (implemented in 1949–1962) reshaped the entire school system, the reform we consider kept the school system relatively intact. Thus, there is much less ambiguity concerning the mechanisms driving the results. Third, the reform was implemented around 70 years ago, implying that mortality outcomes can be studied over a very long time horizon. Fourth, the rate of compliance with the reform was very high: at the time, an overwhelming majority of Swedes would only receive the minimum level of education.

There are a number of empirical studies that use similar methods to uncover the effects of schooling on health. The study most similar in spirit to ours is Lleras-Muney [13], which considers compulsory schooling reforms in the U.S. in the early 20th century. Using a host of compulsory education laws from the 1915–1939 period in instrumental variable (IV) estimations, Lleras-Muney concludes that an additional year of schooling led to reductions in 10-year mortality rates by as much as 60% (six percentage points). This effect is relatively large compared to what has been found in subsequent studies. Clark and Royer [10] study the effects of two British reforms on mortality and a host of other health outcomes. They find that small effects on mortality and coefficients often even have the “wrong” sign. A recent paper by Meghir *et al.* [14] considers the effect of the Swedish 1949–1962 expansion of compulsory schooling on mortality, hospitalizations and labor market transitions. Their estimates for mortality suggest that there was a substantial reduction in male mortality up to the age of 50, but that these gains were erased by elevated mortality at higher ages. Similar patterns are observed for hospitalizations. Another recent paper is Lager and Torssander [15], also considering the Swedish 1949–1962 expansion of compulsory schooling. Separating between death causes, Lager and Torssander [15] find lower mortality in the treated group from death causes generally seen as related to more and better education, such as cancer and accident mortality. Lower mortality is also found among the least educated not continuing to senior secondary or tertiary schooling. The estimates are, however, generally small, and the authors conclude that there does not seem to exist any impact of this later schooling reform on all-cause mortality in all ages.

In an overview of the previous literature, Mazumder [16] concludes that the overall evidence of an effect of education on mortality is weak and that it may have different effects in different countries. Evidence is stronger for the U.S., Germany and the Netherlands than for the UK, Sweden and France. However, the issue of external validity does not only relate to the country context: there may also be important differences between different time periods. Gathmann *et al.* [17] compare reforms from different countries and argue that the most consistent reductions in mortality (for both shorter and longer-run mortality) are found (for men) for a 1919 reform in Belgium and for women for a 1928 reform in the Netherlands. Thus, it appears that earlier schooling reforms have a stronger impact on mortality than those implemented after WWII. To the extent that these differences are down to education having a different effect at different levels of economic development, they imply that our findings are particularly relevant in a low-or medium-income context and not necessarily for inferring the possible effects of further extensions to compulsory schooling in present-day Sweden. Table 1 presents findings, data, and methodological strategies in recent contributions to the literature on the causal effects of compulsory schooling on mortality. It highlights the fact that the institutional setting and the timing of the reform matters.

Our results suggest that the relative reduction in mortality brought by an additional year of compulsory schooling is much larger than the one found by Lleras-Muney [13]. However, the discrepancy appears to be attributable to the higher rate of compliance in Sweden. According to our preferred specification, a reduction in mortality is discernible already before the age of 30. The effect then grows in magnitude and reaches its maximum around the age of 55–60, after which it remains constant or declines slightly. Thus, we do not observe the reversal of effects reported by Meghir *et al.* [14]. Our general findings appear to fit in line with the results shown in Table 1 and the meta analysis of Gathmann *et al.* [17] that reforms implemented in the early 20th century are probably the most effective concerning a reduction in mortality. A major advantage compared to other studies is the possibility that we can actually also estimate short-run effects on mortality for an early implemented reform. The previous studies can usually only fit models conditional on survival up to a certain point in time.

The rest of the paper is organized as follows. Section 2.1 provides a brief history of the Swedish school system, with particular emphasis on reforms during the first half of the 20th century. Section 2.2 gives an overview of the data sources used, and Section 2.4 presents our empirical strategy. Section 3 provides the results, while Section 4 concludes.

**Table 1:** Literature overview: causal effects of compulsory schooling on mortality.

Authors	Country/Data Source	Year/Content of the Reform	Identification Strategy	Main Results
Albouy and Lequien [18]	France/ Longitudinal data: Echantillon Demographique Permanent Census data (1968, 1975, 1982, 1990, 1999) Register Data of Deaths from 1968–2005	1936 (Zay Reform)/6→7 1967 (Berthoin Reform)/7→9	Regression Discontinuity Design on birth cohorts	Zay Reform: survival till 82 for those surviving until 1968 increased by 6% (Wald-estimate). Berthoin Reform: survival until 52 for those surviving until 1968 increased by 1% (Wald-estimate) Effects statistically insignificant
Gathmann *et al.* [17]	Various European Countries/ Human Mortality Database European Social Service International Social Survey Programme Survey of Health Ageing and Retirement	19 different Reforms	Regression Discontinuity Design on birth cohorts Meta analysis (for pooled estimate over the 19 reforms)	Substantial heterogeneity in time and space: Effects probably larger for reforms implemented earlier in the 20th century. Gender differences: no effects for women; reduction of 2.8% in 20-year male mortality from age 18 (reduced form)
Van Kippersluis *et al.* [19]	Netherlands/ Dutch Cross-sectional General Household Survey (1997–2005) Tax Records (1998) Cause-of-Death register (1998–2005) Dutch Municipality Register	1928/6→7	Regression Discontinuity Design on date of birth (individual data)	2%–3% decrease in mortality until the age of 89 for those surviving until the age of 81 (reduced form). Reduced form similar to two-stage least squares estimates as a rise in education between 0.6–1, depending on specification
Clark and Royer [10]	England and Wales/ Mortality Data from the Office for National Statistics: All deaths for the years 1970 to 2007	1947/8→9 1972/9→10	Regression Discontinuity Design	Hardly any evidence for a reduction of mortality; some estimates even with a positive sign
Meghir *et al.* [14]	Sweden Swedish population censuses; all individuals born in Sweden between 1946 and 1957	Implemented by municipalities between 1949 and 1962. From 1962 nationwide/ (7 or 8)→9	Reduced Form Difference in Difference/IV	Short-lived gain in expected male years of life from a shift in mortality from ages 45–50 to ages 50–55. Overall life expectancy not significantly affected Heterogeneity with respect to social background
Lleras-Muney [13]	U.S./ Census (1960, 1970, 1980) National Health and Nutrition Examination Survey	1915–1939 Various U.S. states with different extensions	Difference in Difference/IV Regression Discontinuity Design	Extension of one year of education decreases 10 year mortality for those surviving until 1960 by 3.6% (instrumental variable (IV)) relative to a baseline mortality of 10%. Estimates challenged by Mazumder [16]: Sensitive to state-specific time trends; effects mainly due to earliest cohorts
Lager and Torssander [15]	Sweden/ Swedish population censuses All individuals born in Sweden between 1943 and 1955	Implemented by municipalities between 1949 and 1962 From 1962, nationwide/ (7 or 8)→9	Reduced Form Difference in Difference/IV	Overall, all-cause mortality not significantly affected Lower mortality from causes related to education (e.g., cancer and accidents). Socioeconomic heterogeneity with lower mortality among the least educated

## 2. Institutional Background and Methodological Section

### 2.1. Background on the Educational System and the Reform

Compulsory school attendance was introduced in Sweden in 1842 and applied to all resident children. The compulsory school attendance implied both the right to cost-free schooling in compulsory schools and the obligation to take part in the schooling offered. The central management of the education system was practiced by the *Ecklesiastikdepartementet*, the Ministry for Ecclesiastical affairs. The country was divided into more than 2,000 school districts, and the local administration of compulsory education in these school districts was the responsibility of a school board (education policies were consequently not designed at the county (regional) level, which is the level we use in our empirical analysis). With the exception of girls’ schools, private schools were always insignificant in Sweden [20].

At the beginning of the 20th century, the Swedish educational system was highly selective. Schooling started at the age of seven and was compulsory for six years. The vast majority ended school after these six years in *Folkskolan*. In 1918, it was further decided that sixth grade pupils that did not continue to the non-compulsory secondary school also had to take two years of vocational courses [20]. These were all practically oriented one-or two-year courses in domestic science, craft or manufacturing given at local schools. Importantly, the vocational courses were taught with a very low intensity. As described by Fredriksson [21], the time for vocational courses was only 180 h per year.

Students who chose to follow an academic path continued to lower secondary school to pass the so-called *realexamen* (although rare, students could choose to continue onto lower secondary school already at the age of 11, *i.e.*, leaving compulsory school after the fourth grade, implying that the Swedish educational system had parallel structures). However, secondary schools became widely spread geographically only between the mid-1940s and the early 1960s [22], and very few students continued to secondary schooling before the 1950s. In 1940, only 10% of the cohort graduating from *Folkskolan* continued in secondary school [23]. After realexamen, students either left school or entered the upper secondary level and had to sit in the prestigious *studentexamen* after three more years of studies. This was comparable to the French BAC, German *Abitur* and the English A-levels and a prerequisite for matriculating into university [24]. With a highly selective school system, only 5% of a cohort continued to upper secondary schooling in 1940 [23].

Students in *Folkskolan* were attending full-time schooling approximately eight months per year. In the rural areas, it was, however, also possible for school districts to offer half-time reading, so that children attended school only certain days of the week. This school form only existed in the rural areas of Sweden, where children often helped out in the agrarian sector. The existence of half-time reading was heavily debated in the 1920s, and the extent of half-time reading was reduced during this decade, although still permitted. In 1933, 93% of all pupils took part in full-time schooling [21].

For a long time, local conditions decided the format and content of primary education, and at the beginning of the 20th century, there was large variation across school districts. The national government issued its directives for the curriculum in so-called *normalplaner* (normal plans), but this document was only advisory. In 1919, however, the so-called *Utbildningsplanen* (the education plan) was introduced, which came to restructure the school’s work according to the central guidelines of Ecklesiastikdepartementet. Utbildningsplanen was a governing document and included time-tables and syllabuses for compulsory school [25]. It is, of course, difficult to know about the quality of the education across school districts. Completion rates were, however, high: 90% of all pupils finished Folkskolan with full curriculum [21]. The implementation of the Utbildningsplan was the first instance in which local autonomy had to give way for national standards, and the Government’s edict was subject to financial compensation [20].

On 1 July 1936, the national Government decided that seven-year school should be compulsory. Already, in 1925, a clause had been introduced in the primary school code that a seventh school year could be made compulsory [21]. School districts were also allowed to introduce eight year compulsory schooling, but this was a very rare event, both in urban and rural areas. In 1940, only 0.1% of all schools in the country offered eight years of education [21]. To extend compulsory schooling with an extra year was then the decision of the school board in a school district, and already, in 1936, several school districts in the urban areas had introduced an extra year of schooling. Furthermore, in the southernmost and mainly rural region of Scania, several school districts had implemented seven years of schooling, but, still, only 16% of all children in rural areas of Sweden attended seven years of schooling in 1936.

In Sweden, child labor laws and compulsory attendance laws have generally been coordinated. One basic principle has been that the right to education takes precedence over the demands of the labor market—so that educational requirements with respect to knowledge and time should determine if and when the young were allowed to work. Compulsory school attendance regulations have consequently reinforced the protective labor legislation, and as discussed by Sjöberg [26], Swedish authorities have generally relied on double protection—age limits and compulsory school attendance. According to the 1931 Labor Act, the minimum age for manufacturing and construction work was 14 years, whereas the limit for “light work” was 13 years. A child was allowed to work from the beginning of the calendar year in which they would reach the age limit. After the implementation of the compulsory school reform, most pupils left school in the middle of the year they turned 14, whereas before they would have left the year, they turned 13. This means that the reform reduced the time a child could spend in “light work” by one year, whereas the corresponding reduction for “hard” (industrial) work would have been 5–6 months only. The 1949 Labor Act increased the age limit by one year, in turn harmonizing the age when a majority of students finished schooling and started to work. Notably, the legal documents generally regulated full-time work, but not the part-time work of young people [26].

The main motive for the reform was that six years was considered too short for achieving the learning objectives that were stated for the *Folkskolan*. This motive was also mentioned in the decision allowing school districts to implement seven years of compulsory schooling in 1925. Additional arguments on the importance of a change in the compulsory schooling legislation mentioned in various investigations were the increasing youth unemployment among those who had just finished elementary school, but also that another year of education was of importance to maintain a democratic society [20]. In the debate preceding the introduction of the reform, politicians were also often benchmarking with other Western countries, and it was noted that the number of school years was the most striking difference of compulsory education in Sweden compared with Denmark, Norway, Germany and Great Britain.

In line with the underlying motive for the parliamentary decision, the reform did not require any fundamental changes with respect to learning outcomes to be achieved or curricula, but instead emphasized the goal of achieving more long-lasting results of schooling. The recommendation from the central administration was that the school districts should distribute the pre-reform compulsory school curricula over seven years instead of six [20].

**Figure 1:**
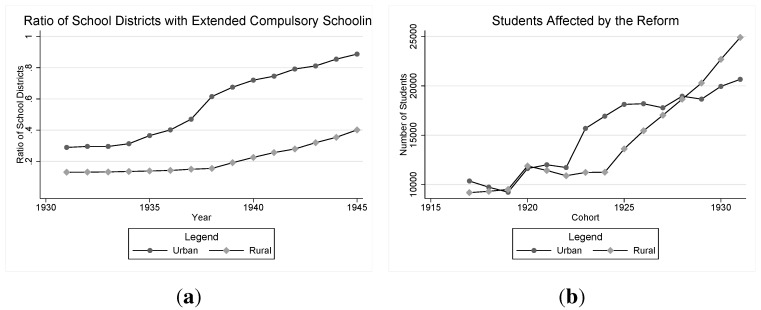
(**a**) Proportion of school districts with seven years of compulsory schooling; (**b**) number of students affected.

The reform was not implemented at the same time in all school districts, but, instead, it was stipulated that it had to be implemented in all school districts before 1949. The compulsory seventh year was consequently introduced during a twelve-year transition period. In 1936–1941, an implementation of an extra year was completely at the discretion of the school district. From 1942 and onwards, a school district could be assigned to implement the reform, but according to official sources, this only happened once. The national school authority *Skolöverstyrelsen*, monitored the implementation. Initially, the reform led to a relatively rapid transition. In 1940, 33% of the rural and 80% of the urban schools had implemented a seventh year [21]. After 1940, the implementation rate, however, seems to have decreased somewhat. Figure 1(a) shows the trends in implementation and reports the proportion of school districts that had at least seven years of compulsory schooling at the end of each school year, and Figure 1(b) shows the number of students affected by the reform by birth cohort.

Due to the soft transition rules, the reform does not seem to have caused any major difficulties in the school districts, and the implementation was also facilitated by the fact that the responsibility for funding of school buildings, teaching materials and teachers’ salaries was the responsibility of the central government and not the school districts [27].

From 1936 until the 1950s, not much else other than the implementation of the seven year reform changed in the Swedish educational system. In 1948, a parliamentary school committee proposed a new school reform that implied nine-year compulsory and comprehensive schooling. Additionally, this reform, starting in 1949 and evaluated, e.g., in Meghir and Palme [28] and Meghir *et al.* [14], was gradually implemented across school districts over time. The major consequences of this reform were additional years of schooling, but also a reshaping of the entire school system.

As regards the wider institutional context, we are unaware of any reform relevant to public health that might have coincided with the school reforms at the local level. Such a coincidence is unlikely, since decisions related to healthcare were taken at either the county level or at the health district level, both of which typically consisted of several school districts. Moreover, most of the expansion of the Swedish healthcare system came after the introduction of uniform social health insurance in 1955 [29]. Thus, access to services was limited and did not change much during the period we consider. For example, a survey made by the National Board of Health (*Medcinalstyrelsen*) in 1927 concluded it was very rare that pregnant women had been examined by any kind of medical staff, including midwifes, before delivery [30]. This lack of coverage was perceived as a problem and led to a national roll-out of preventative services for pregnant women and newborns from 1937 onwards [31]. Moreover, as discussed by Heidenheimer *et al.* [32], the physician density in Sweden was significantly lower compared to, e.g., Norway and Denmark, in the 1930–1950 period, and official statistics suggest that the number of hospitals and hospital beds *per capita* remained more or less constant [33]. One trend that did, however, affect the cohorts included in this study was the transition to institutional delivery, which became the standard between the years 1920–1940 [34]. Even though this general trend could theoretically confound our analysis, it is very unlikely that it does so in practice. The expansion in hospital births was very smooth and mainly driven by individual-level demand-side decisions.

Another potential concern relates to World War II (1939–1945) and the fact that compulsory schooling generally protects individuals from getting involved in armed conflicts. If Swedes joined armed forces and died in battle, this could generate a misinterpretation of the results as an effect of education, while rather capturing an education gradient in enlistment. Notably, however, Sweden was neutral in World War II, and less than 9,000 individuals (almost all of them men and all older than 18 years) fought in the war as volunteers [35], suggesting this should be a minor problem in our analysis.

In summary, the reform in 1936 introduced an exogenous change in the extent of compulsory schooling in Sweden. The timing of implementation in individual school districts was based on a mixture of local and national decisions; and there is no reason to suspect that the implementation of the reform was driven by health differences. Thus, it will be our working assumption that the reform was exogenous from the individual point of view so that it can be used to identify the effects of schooling on health. As regards the exact definition of treatment, the above account has made clear that affected pupils faced no significant increase in the curriculum to be covered. Thus, effects, if present, will be driven by changes in the amount of time spent in education.

### 2.2. Data and Sample Selection

We combine three different datasets in our analysis. Our school reform indicator is based on unpublished material taken from the archive of the national school authority [36]. After the national government had decided to make a seventh year compulsory in 1936, the national school authority, *Skolöverstyrelsen*, monitored the implementation. Data are available from 1931 onwards for rural areas and from 1938 onwards for urban areas. These tables were based on reports from individual school districts, and they provide the number of districts that had introduced seven (and in some rare cases, eight) years of schooling. In a small number of cases, the classification of districts was challenging, since the seventh year was introduced at different times for different schools within the district. In the original data, such districts have been coded as having seven years of schooling. Given the low number of school districts where this is an issue, this is unlikely to lead to measurement error.

A more serious concern is that we do not have the *identity* of the school districts that have extended compulsory schooling—only in exceptional cases (*i.e.*, when there is only one city in the county) is it possible to unambiguously identify the reform year. Thus, we use the proportion of reformed districts as our treatment variable.

Our second source of data is the census of 1935 [37]. Traditionally, Sweden had a complete census every ten years; however, in 1935 and 1945, there were additional censuses, which provided basic demographic information on the whole population and, then, studied some parts of the country in more detail. The 1935 census gives us information on cohort sizes, which are used for calculating mortality rates at different ages. For each of the 25 counties, we collect cohort sizes of males and females from urban and rural areas separately—the exception being the City of Stockholm, which was the only county without rural areas. Thus, the total number of cells in each year is 98.

Our third data source consists of the Swedish Death Index, which is a digital data source provided by Federation of Swedish Genealogical Societies [38]. The dataset is based on official records, such as church books, and it is complete from 1947 onwards. For earlier years, 25% of deaths are missing at random throughout the country—with the sole exception of the county of Värmland, which is complete. Since our cohort size data give cohort sizes after infancy, the missing deaths, thus, apply to ages at which mortality rates approach their lifetime minimum. For the cohorts included in our dataset, the cumulative mortality rates between the baseline year and the first year with complete records (*i.e.*, for the period 1936–1946) vary between 1.2% and 2.2%, according to the life tables of the period (our calculations from the census tables). Therefore, the measurement error due to the missing information is likely to be small, but in order to correct for it we multiplied annual cohort-specific mortality rates in relevant years by a factor of 1.33 (*i.e.*, the factor necessary to compensate for the missing deaths).

The Swedish Death Index contains information on gender, date of birth, date of death, parish of birth and parish of death. Since all parishes can be attributed to either the urban or the rural category (and some of them were recategorized during the time covered), we can add them up for each region to get the numerator of the mortality rate. The Swedish Death Index has the advantage that we are able to calculate death rates for the short run, as we can practically observe all deaths in the whole of Sweden for each year.

However, a slight complication arises, due to the coding of birth parishes. Prior to 1946, it was common to attribute hospital births to the place of birth (*i.e.*, the hospital parish) and not to the place of residence (*cf*., [39]). This is a serious concern for cohorts born in the 1940s, when institutional delivery became the norm. However, during the period we consider, hospital births were still exceptional and, also, predominantly affected children from the cities. Consequently, the measurement error arising is limited, but we circumvent this problem by aggregating urban and rural areas in our baseline specification—which brings the number of cells down to 50 per year.

### 2.3. Variable Definitions

We now turn to a detailed description of how the main variables in our analysis have been constructed. We start with our outcome variable—mortality rates at different ages—and, then, turn to the school reform variable. Summary statistics of all main variables are provided in Table 2.

**Table 2:** Summary statistics.

	**Mean**	**Standard Error**	min	max	***N***
**10 Year Death Rate**	0.007	0.004	0.000	0.027	731,791
**20 Year Death Rate**	0.017	0.007	0.000	0.047	731,791
**30 Year Death Rate**	0.026	0.009	0.004	0.073	731,791
**40 Year Death Rate**	0.048	0.016	0.010	0.111	731,791
**50 Year Death Rate**	0.097	0.035	0.025	0.218	731,791
**60 Year Death Rate**	0.193	0.069	0.050	0.427	731,791
**70 Year Death Rate**	0.373	0.118	0.105	0.746	731,791
**Male**	0.509	0.500	0.000	1.000	731,791
**Treatment**	0.406	0.272	0.000	1.000	731,791
**Urban**	0.269	0.223	0.040	1.000	731,791
**Age at Census 1935**	7.622	2.299	4.000	11.000	731,791
***G***	400				

***G*** corresponds to the number of cells defined by gender, cohort and county of birth. All statistics are calculated using weights. Weights are given by the number of observations in each cell.

#### 2.3.1. Computing Mortality Rates

As mentioned above, we calculate mortality rates before certain ages combining two data sources, the Swedish Death Index and the 1935 census. The population at risk is defined by cohort-county-gender-urban cells for the cohorts 1924–1931—who were between four and 11 years old at the time of the census. The number of cohorts we can include is restricted by our treatment variable—which is complete only for the years 1938–1945. The choice of the census enables us to count individuals before they finish compulsory schooling. Therefore, we can circumvent moving induced by the treatment of one more year of schooling, a factor that appears possible, as education is often associated with mobility. The choice of using the census, however, lead to the possibility that treatment-induced selection into regions with better healthcare access are picked up as part of the treatment.

The mortality rates have been calculated as *x*-years mortality rates for each cohort-county-gender cell:

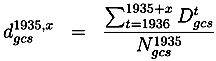

where 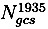 denotes the size of cohort *c* of sex *s* in county *g* at the end of 1935 and 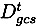, the number of deaths of the corresponding cell during year *t*. The incomplete deaths between 1935 and 1946 have been imputed under a *missing at random* assumption. The imputed death rates roughly match death rates from official statistics. Computing death rates from a later census to circumvent the missing values in the Death Index was not possible, due to migration into cities. The cell-specific population in 1950 deviates from the population 20 years earlier. This would be problematic with respect to the treatment assignment. As students in cities are more likely to have experienced extended schooling, many more individuals would be assigned as treated if a later census would have been used.

#### 2.3.2. Treatment Indicator

As mentioned above, information to construct the treatment indicator was taken from Skolöverstyrelsen [36]. There were more than 2,300 rural school districts and around 120 urban school districts in total. If we denote by *q_gt_* the number of school districts in region *g* that had extended compulsory schooling in year *t* and the total number of school districts in the same year as *Q_gt_*, we may define our treatment indicator as a fraction:

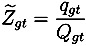
(1)


**Figure 2:**
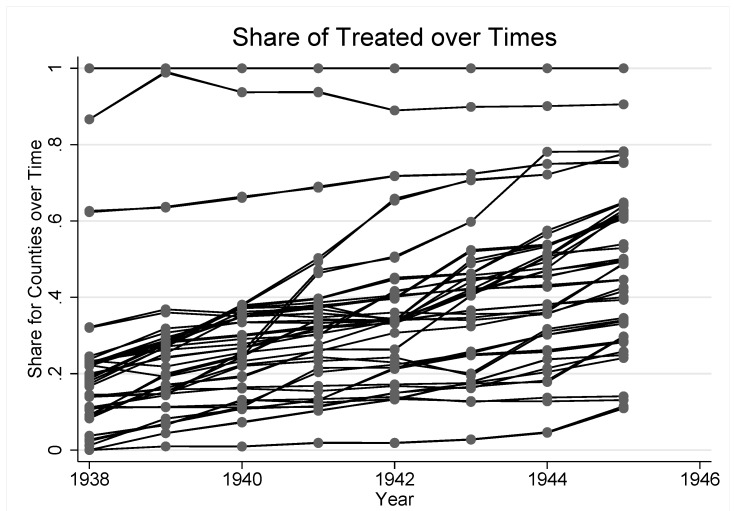
Share of treated school districts.

If the variation in school district size within a county is relatively small, this variable will approximate the proportion of individuals in county *g* who are exposed to compulsory schooling beyond the age of 13 in year *t*. Thus, in order to get the assignment of the treatment variable right, we need to take into account that the cohort, *c*, which started school in the fall of year *c* +7, will be affected by the extension of compulsory schooling in year *c*+13, and if so, they may leave school no earlier than year *c*+14. Thus, cohort *c* will be exposed to the treatment indicator, 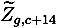. As we combine rural and urban areas for reasons mentioned in Section 2.2, the final treatment share is constructed as a weighted average between the two areas in every region, *g*. The weights are given by the specific cohort sizes from the census. In Figure 2, we have plotted 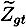 for each region.

### 2.4. Empirical Strategy

In our basic specification, the probability of dying within the next *x*-years from 1935 for an individual 
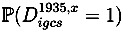
 is assumed to be given by a linear probability model:

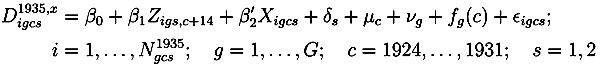
(2)
where *c* indicates the cohort, *g* the region, *Z_igs,c_*_+14_ is an indicator of whether an individual has been affected by the extension of compulsory schooling, *X_isgc_* is a vector of covariates, including a dummy of whether an individual resides in an urban area in 1935, *µ_c_* and *ν_g_* are fixed cohort and regional effects, *δ_s_* is a gender-specific constant and *f_g_*(*c*) is a region-specific cohort trend. Given the small number of cohorts, we approximate the regional trend by polynomials of the first and second order. 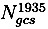 gives the number of observations within a specific gender-cohort-region cell. In the basic regressions without the inclusion of cohort trends, *β*_1_ is identified by deviations from a statewide cohort trend and regional specific intercepts and, therefore, constitutes a simple difference-in-difference estimate. This specification has been shown to be sensitive concerning the inclusion of regional specific trends (see Mazumder [16]).

As mentioned before, we do not observe the treatment indicator for the individual, but only the share of schools with extended compulsory schooling within a region, *g*, for a specific cohort, *c*. Averaging Equation (2) over the observations in each gender-cohort-region cell produces:

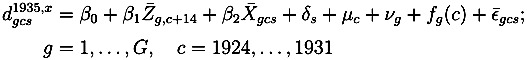
(3)


The treatment is now given by the ratio of students affected by extended compulsory schooling, 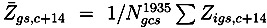. In general, the constructed treatment share, 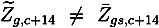, as 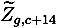, does not distinguish between gender and weighs all school districts equally by construction. This creates a (possibly non-classical) measurement error in the instrument. In the following, we will carefully state the assumptions that are necessary for inference on the effects of the treatment, *β*_1_.

Assuming (conditional) random assignment of extended compulsory schooling to school districts (or schools) within counties, the measurement error in our instrument has the expectation of zero. The estimates then represent a lower bound for *β*_1_. This is unproblematic with respect to the gender aggregation. However, as we do not know the size of the school districts, non-random assignment with respect to the number of students could occur. Larger districts could, for example, introduce the extended minimum schooling first. If the state trends and the covariates insufficiently control for this possibility, the measurement error would not necessarily lead to an attenuation bias. Instead, the sign of the bias is unclear.

Most importantly, inference on individual behavior, as described by Equation (2), is achieved from aggregates. Therefore, we are subject of committing an *ecological fallacy*. The problems associated with inferring individual behavior from aggregates is well-known Robinson [40] (in his seminal work, Robinson investigated the relationship between the ratio of foreigners and the literacy rate in U.S. states. The positive relationship on the aggregate level is a spurious correlation from foreigners living mostly in areas with higher literacy rates and disappears if confronted with individual data (Freedman [41])). The problem occurs, as the (conditional) joint distribution of the mortality rates and the treatment is the aim of inference, while only the marginal distributions are observed. We do not know the exact combinations of mortality and schooling for each individual. This makes regressions on aggregate levels especially prone to spurious results.

Without imposing strong assumptions, it is not possible to draw conclusions about the joint distribution using only marginal distributions from aggregated data. Though this issue cannot be solved without individual level data, the reliability of the estimates can be nevertheless strengthened. Given that we use variation between cohorts within a county, we can reasonably correct, to some extent, for possible confounding. Prior studies indeed confirmed that the aggregation bias can be sufficiently relaxed in this way. For example, Lleras-Muney [13] uses the same specification to model the effects of education on mortality rates. Comparing analysis from individual survey data and aggregated data from official statistics, her results only differ slightly. This indicates the absence of a serious aggregation bias.

Note that we face only partial compliance, as not all students change their educational decision, due to an extension of the years of compulsory schooling. Therefore, Equation (2) can be interpreted as a reduced form for the effects of education on mortality. Under random assignment of the instrument and the absence of an aggregation bias, the estimation yields a lower bound for an intention to treat a parameter of compulsory schooling extensions. It is a lower bound, due to the measurement error in the share of districts treated relative to the number of students.

In Meghir and Palme [28], for cohorts born between 1940 and 1957, the share of fathers having more than compulsory schooling can be found. These fathers could potentially be treated in our application. The proportion of men having more than compulsory schooling is less than 25% in their sample. This would indicate a large compliance rate for the reform. Without the reform, it is very likely that most of the individuals would have experienced less schooling. The estimates should, therefore, be close to the effects on those kept longer in school, only due to the extension of compulsory schooling.

## 3. Results and Discussion

Under valid assumptions, the estimates yield a conservative bound for the direct effect of the changes in compulsory schooling on mortality rates. Several *x*-year death-rates are regressed on the share of school districts within a county treated by an extended compulsory schooling. Table 3 presents the results of our main specification, which includes cohort dummies, county dummies and time trends (bottom part). Additional specifications have been included in Table 4 to assess the robustness of the baseline results.

All specifications indicate a reduction of mortality rates by extended minimum years of schooling. Adding possible confounders, such as state effects, the share of urban population within a county of the gender composition does not qualitatively change the result. However, with respect to the relevant baseline mortality, the magnitude of the effects appears rather large. Estimating the effects of the treatment on the 30-year death rate controlling for cohort and county dummies, as well as urban status and gender leads to a reduction of about 30% at the mean. Further, inserting linear trends even increases the effect size. Given the greater flexibility, we prefer the specification with linear trends. The additional variation explained is low. The effects from compulsory schooling are, nevertheless, for some death rates sensitive to the inclusion of the state-specific trends. Standard errors are clustered on the county-cohort level and are robust against heteroscedasticity—which is inherent in the linear probability model.

It should be noted that our results cannot be directly linked to the effects arising from increases in years of schooling, as we only observe compulsory schooling, but not education itself. Comparing our results to the reduced form estimates in Lleras-Muney [13] using the same identification strategy, but without measurement error in the instrument, the estimates for the 10-year death rates are of comparable size. However, in stark contrast, Lleras-Muney’s 10-year death rates refer to a much older population. The baseline mortality is much larger for her sample, indicating much smaller *relative* effects.

**Table 3:** Estimation results: main specification.

**Death Rate:**	10 Years	20 Years	30 Years	40 Years	50 Years	60 Years	70 Years
**No trends:**							
**Treatment**	−0.004*	−0.006*	−0.008*	−0.011**	−0.010*	−0.013	−0.028*
	(0.002)	(0.002)	(0.003)	(0.004)	(0.005)	(0.008)	(0.013)
**Urban**	−0.004	−0.015	−0.026	0.001	−0.009	−0.086	−0.034
	(0.012)	(0.018)	(0.021)	(0.028)	(0.051)	(0.083)	(0.111)
**Male**	0.002**	0.006**	0.012**	0.022**	0.051**	0.102**	0.164**
	(0.000)	(0.000)	(0.000)	(0.001)	(0.001)	(0.002)	(0.002)
***R^2^***	0.659	0.734	0.827	0.894	0.933	0.951	0.973
**Linear county trends:**							
**Treatment**	−0.005	−0.014**	−0.025**	−0.023**	−0.029**	−0.016	−0.026
	(0.003)	(0.005)	(0.006)	(0.008)	(0.010)	(0.012)	(0.019)
**Urban**	−0.003	−0.010	−0.015	0.008	−0.001	−0.092	−0.012
	(0.012)	(0.019)	(0.022)	(0.030)	(0.054)	(0.086)	(0.115)
**Male**	0.002**	0.006**	0.012**	0.022**	0.051**	0.102**	0.164**
	(0.000)	(0.000)	(0.000)	(0.001)	(0.001)	(0.002)	(0.002)
***R^2^***	0.682	0.760	0.848	0.901	0.936	0.954	0.975
**Linear/Quadratic county trends:**							
**Treatment**	−0.008*	−0.020**	−0.028**	−0.023*	−0.034**	−0.024	−0.019
	(0.004)	(0.005)	(0.008)	(0.009)	(0.011)	(0.013)	(0.021)
**Urban**	−0.005	−0.006	−0.014	−0.002	0.001	−0.099	−0.039
	(0.014)	(0.020)	(0.025)	(0.033)	(0.060)	(0.095)	(0.124)
**Male**	0.002**	0.006**	0.012**	0.022**	0.051**	0.102**	0.164**
	(0.000)	(0.000)	(0.000)	(0.001)	(0.001)	(0.002)	(0.002)
***R^2^***	0.705	0.773	0.856	0.907	0.939	0.957	0.977
***G***	400	400	400	400	400	400	400
**Baseline Mortality**	0.007	0.017	0.026	0.048	0.097	0.193	0.373

Dependent variable: average number of deaths within x-years. Additional controls: cohort dummies, county dummies. Standard errors (in parentheses) are robust against heteroscedasticity and clustered on the cohort state level. ***G*** corresponds to the number of cells defined by gender, cohort and county of birth. All regressions are calculated using weights. Weights are given by the number of observations in each cell.

* signifies that the relationship between the point estimate and standard error is above the level required for statistical significance at the 10% level in a linear regression model, ^**^ at the 5% level and ^***^ at the 1% level.

**Table 4:** Estimation results: different specifications.

**Specification:**	(1)	(2)	(3)	(4)	(5)	(6)	(7)	(8)	(9)	(10)	(11)
**Death Rate:**											
**10 Years**											
** Treatment**	−0.001	−0.004*	−0.004*	−0.004*	−0.004*	−0.006	−0.005	−0.005	−0.005	−0.007*	−0.007*
	(0.001)	(0.002)	(0.002)	(0.002)	(0.002)	(0.003)	(0.004)	(0.003)	(0.003)	(0.003)	(0.003)
**20 Years**											
** Treatment**	−0.003*	−0.006**	−0.006*	−0.006**	−0.006*	−0.016**	−0.014**	−0.015**	−0.014**	−0.016**	−0.016**
	(0.001)	(0.002)	(0.002)	(0.002)	(0.002)	(0.005)	(0.005)	(0.005)	(0.005)	(0.005)	(0.004)
**30 Years**											
** Treatment**	−0.004**	−0.008**	−0.008*	−0.009**	−0.008*	−0.029**	−0.026**	−0.027**	−0.025**	−0.028**	−0.028**
	(0.002)	(0.003)	(0.003)	(0.003)	(0.003)	(0.006)	(0.006)	(0.006)	(0.006)	(0.006)	(0.005)
**40 Years**											
** Treatment**	−0.006	−0.012**	−0.011**	−0.012**	−0.011**	−0.030**	−0.023**	−0.027**	−0.023**	−0.026**	−0.026**
	(0.003)	(0.004)	(0.004)	(0.004)	(0.004)	(0.008)	(0.008)	(0.008)	(0.008)	(0.008)	(0.008)
**50 Years**											
** Treatment**	−0.009	−0.013*	−0.010*	−0.014*	−0.010*	−0.045**	−0.029**	−0.037**	−0.029**	−0.029**	−0.030**
	(0.008)	(0.005)	(0.005)	(0.006)	(0.005)	(0.011)	(0.010)	(0.011)	(0.010)	(0.010)	(0.010)
**60 Years**											
** Treatment**	−0.018	−0.017*	−0.012	−0.020*	−0.013	−0.052*	−0.018	−0.033	−0.016	−0.022	−0.022
	(0.016)	(0.008)	(0.008)	(0.010)	(0.008)	(0.020)	(0.012)	(0.018)	(0.012)	(0.012)	(0.012)
**70 Years**											
** Treatment**	−0.036	−0.035*	−0.028*	−0.040*	−0.028*	−0.080*	−0.026	−0.054	−0.026	−0.031	−0.029
	(0.028)	(0.014)	(0.013)	(0.016)	(0.013)	(0.034)	(0.020)	(0.032)	(0.019)	(0.019)	(0.018)
**Urban**				√	√			√	√	√	√
**Male**			√		√		√		√	√	√
**County FE**		√	√	√	√	√	√	√	√	√	√
**Cohort FE**		√	√	√	√	√	√	√	√	√	√
**County trends (linear)**						√	√	√	√	√	√
**Gender trend**										√	
**Restricted sample**											√
***G***	400	400	400	400	400	400	400	400	400	400	368

Standard errors (in parentheses) are robust against heteroscedasticity and clustered on the cohort state level. ***G*** corresponds to the number of cells defined by gender, cohort and county of birth. All regressions are calculated using weights. Weights are given by the number of observations in each cell. The gender trend is a cohort gender interaction. Specification (11) drops *Stockholm Stad*, *Uppsala län* and Stockholm län. * signifies that the relationship between the point estimate and standard error is above the level required for statistical significance at the 10% level in a linear regression model, ** at the 5% level and *** at the 1% level.

In comparing the results, one has to keep in mind that the compliance rate in our case is much higher. If nobody in the population would experience more schooling than compulsory and the laws are effective, an extension by one year would ultimately lead to an increase in education of about one year. In Lleras-Muney [13], compulsory schooling laws only raised education by about 0.045 years. Similarly, also Oreopoulos [11] finds relatively small effects of about 10% on education by the extension of compulsory schooling for the U.S. This either means that the laws have been ineffective—or many individuals would have extended schooling anyway. Given the high rate of individuals only receiving compulsory schooling in our data, we assume that the effects of the years of education are much larger. If the extension of the compulsory schooling raises education by slightly less than one year, then the effects of the reduced form estimated here are more comparable with the instrumental variable (IV) estimates in Lleras-Muney [13]. The IV estimates of the effects of education are instead of comparable size: Lleras-Muney observes a relative reduction of 60% from a baseline mortality rate of 10%; we get a 57–71% reduction in 10-year mortality.

Results for the long-run death rates give more plausible effect sizes. They also appear more robust with respect to the inclusion of linear county trends. Given the high mean 70-year death rate, the compulsory schooling extensions reduces mortality risk at the mean of approximately 7% in the specification with trends. Different specifications listed in Table 4 give similar results. The results are sensitive to the inclusion of county-specific trends, which tend to increase the effect sizes. Differentiating the overall trend by gender did not alter the results. Furthermore, three counties have been dropped in the last specification. Due to a transition of parishes between the city of Stockholm, Stockholm County and Uppsala County in 1971, death rates are incorrectly measured. However, omitting the three did not alter the results.

In Table 5, we further investigated treatment effect heterogeneity by gender with a parsimonious parametrization and by splitting the sample. Neither interacting only the treatment and the constant by gender nor estimating the whole model separately for men and women changes the general results. Given that the separated model can be seen as a fully interacted regression, the decrease in precision of the estimates is due to the additional amount of parameters estimated.

As an additional robustness check, Table 6 shows the estimates for a logistic regression. Due to the binary structure of the occurrence of death for the individual, the model is inherently nonlinear. The same applies for the shares in the aggregated regressions, which are bounded between zero and one. Addressing the nonlinearity by estimating a logistic regression instead of a linear model, however, does not change the results. The precision of the estimates is very similar, and marginal effects do not differ to a large extent from the linear specification.

As regards the mechanisms that might drive the results, the data at hand do not allow for a very detailed analysis. However, two striking results which appear to be relatively robust are that (a) most of the effect appears already within the interval of 10–20 years—*i.e.*, between ages 14 and 31, and (b) there are no striking differences between males and females, even if the effect seems to operate at slightly more advanced ages for females. It is thus useful to investigate the leading death causes for males and females in the relevant ages. According to death cause statistics from 1940 [42], the leading death cause in the relevant age group was infectious disease for males and females alike. This death cause was responsible for 40% of deaths for males and more than 50% of deaths for females. The second most prevalent death cause was external causes, which account for 20–30% of male deaths and 6–7% of female deaths, and the majority of deaths within this category are due to accidents, in particular, drowning. Thus, it is indeed plausible that some of the effect is driven by compulsory schooling laws protecting young individuals from a risky working life. However, as was explained at the outset, the entry into physically demanding work was delayed by at most six months, due to the reform. Hence, it seems quite unlikely that this mechanism is the dominating one. Indeed, if the effect is related to occupational hazards, a more plausible mechanism appears to be a treatment-induced selection into physically less demanding jobs.

**Table 5:** Estimation results: gender differences.

	**Death Rate:**	10 Years	20 Years	30 Years	40 Years	50 Years	60 Years	70 Years
**Specification:**								
**Interacted**								
	**No trends:**							
	**Treatment**	−0.004* (0.002)	−0.005* (0.002)	−0.007* (0.003)	−0.011** (0.004)	−0.010 (0.006)	−0.012 (0.011)	−0.031* (0.015)
	**Interaction**	−0.000 (0.001)	−0.001 (0.001)	−0.002 (0.002)	0.000 (0.002)	−0.001 (0.005)	−0.003 (0.009)	0.007 (0.010)
	**Linear county trends:**							
	**Treatment**	−0.005 (0.003)	−0.013** (0.005)	−0.024** (0.006)	−0.023** (0.007)	−0.028** (0.010)	−0.014 (0.013)	−0.029 (0.020)
	**Interaction**	−0.000 (0.001)	−0.001 (0.001)	−0.002 (0.002)	−0.000 (0.002)	−0.001 (0.005)	−0.003 (0.009)	0.006 (0.010)
**Men Only**								
	**No trends:**							
	**Treatment**	−0.003 (0.002)	−0.008* (0.003)	−0.011* (0.004)	−0.013* (0.005)	−0.013 (0.007)	−0.010 (0.011)	−0.030 (0.019)
	**Linear county trends:**							
	**Treatment**	−0.010 (0.005)	−0.024** (0.007)	−0.029** (0.010)	−0.020 (0.013)	−0.026 (0.015)	−0.012 (0.021)	−0.040 (0.028)
**Women Only**								
	**No trends:**							
	**Treatment**	−0.004 (0.002)	−0.003 (0.003)	−0.005 (0.004)	−0.009 (0.005)	−0.008 (0.006)	−0.016 (0.010)	−0.025 (0.013)
	**Linear county trends:**							
	**Treatment**	0.002 (0.006)	−0.004 (0.007)	−0.021** (0.008)	−0.028** (0.011)	−0.032* (0.014)	−0.019 (0.018)	−0.011 (0.025)

Dependent variable: average number of deaths within x-years. Additional controls: cohort dummies, county dummies. Standard errors (in parentheses) are robust against heteroscedasticity and clustered on the cohort state level. All regressions are calculated using weights. Weights are given by the number of observations in each cell. * signifies that the relationship between the point estimate and standard error is above the level required for statistical significance at the 10% level in a linear regression model, ** at the 5% level and *** at the 1% level.

**Table 6:** Logistic regression: main specification.

**Death Rate:**	10 Years	20 Years	30 Years	40 Years	50 Years	60 Years	70 Years
**No trends:**							
**Treatment**	−0.584*	−0.354**	−0.334**	−0.278**	−0.167*	−0.115*	−0.142*
	(0.243)	(0.128)	(0.119)	(0.078)	(0.064)	(0.055)	(0.058)
**Marginal effects**	−0.004	−0.005	−0.008	−0.012	−0.014	−0.017	−0.031
**Linear county trends:**							
**Treatment**	−0.473	−0.764*	−1.037**	−0.565**	−0.350**	−0.105	−0.121
	(0.586)	(0.300)	(0.239)	(0.177)	(0.121)	(0.079)	(0.085)
**Marginal effects**	−0.003	−0.012	−0.026	−0.025	−0.030	−0.016	−0.027
***G***	400	400	400	400	400	400	400

Dependent variable: logistic transformation of the average number of deaths within x-years. Additional controls: cohort dummies, county dummies. Standard errors (in parentheses) are clustered on the cohort state level. ***G*** corresponds to the number of cells defined by gender, cohort and county of birth. All regressions are calculated using weights. Weights are given by the number of observations in each cell. ^**^ at the 5% level and ^***^ at the 1% level.

Given the relatively strong assumptions we had to impose, due to the data limitations, we tend to be very cautious in interpreting the results as clear evidence that compulsory schooling extensions in the beginning of the 20th century in Sweden reduced mortality risk. Given the direction of the results and their robustness to changes in the empirical setup, we nevertheless think that they can be seen as a first hint that effects do exist, even if the effect sizes might not be taken at face value from the aggregate regressions. Our findings should be corroborated with studies using district-level reform information and individual-level data.

## 4. Conclusions

Theoretically, there are several factors suggesting education will have a positive effect on health outcomes: education may improve an individual’s economic situation, enabling health investments and health-related consumption, and education likely also affects health behavior by increasing knowledge or affecting time preferences. Furthermore, empirically, there is a well-known association between education and a wide range of health outcomes, but there is still disagreement with respect to the causal effects of education on health. To some extent, this ambiguity relates to the variety of health outcomes that have been used in previous analyses, but also to methodological challenges and poor identification strategies. Moreover, existing evidence mainly uses information on educational changes quite recent in time, implying that health outcomes are studied over a short time horizon.

We study the mortality effects of a nationwide Swedish reform, where compulsory schooling in primary school was extended by one year in 1936, from six to seven years of schooling. The reform was implemented at the school district level over a transition period of 12 years from 1936–1948. Taking advantage of the general variation in the timing of the implementation of the reform across school districts, we believe it is possible to identify health effects of the reform.

Using data from three different data sets: information on the number of school districts that had introduced seven years of schooling and from the national school authority, the census of 1935 covering all people in the country and the Swedish Death Index, we estimate the effect of the proportion of reformed districts on various crude death rates. Results clearly suggest a robust reduction of mortality rates by extended schooling. Baseline findings come out significant and rather large in magnitude, but controlling for cohort and county dummies, urban/rural status and gender reduce the effect sizes. Considering long-run death rates also gives plausible effects.

Our results are quite similar to existing studies using similar methods to uncover the effects of schooling on health (see, e.g., [10,13]). A recent study by Meghir *et al.* [14] examines the mortality effect of a later expansion of compulsory schooling in Sweden in 1949–1962—a school reform not only extending the compulsory number of years in school, but also abolishing tracking in secondary school and vocational courses. They find that additional education reduces male morality up to the age of 50, but that mortality is elevated at later ages—a reversal effect we do not find when analyzing the seven-year compulsory school reform.

Indeed, our results suggest that increases in compulsory education have a large and persistent effect on survival prospects. For example, we estimated that the 50-year survival rate increased by 3.2% due to the reform. A simple back-of-the envelope calculation suggests that 0.8 life years were gained during this period. Thus, considering the fact that the reform only affected those who left school at the earliest possible age, the treatment effect on the treated may well have been an extension to their life expectancy, which goes beyond their extension of schooling. The causal effect of education and schooling might thus explain a large chunk of the difference in life expectancy between educational groups that was reported in the introductory Section.

In the time period studied, very few students continued to secondary schooling, and compliance with the reform was very high. The negative effect on mortality thus suggests that the true effect is partly mediated by the extra year of compulsory schooling itself, although our results rely on relatively strong assumptions, due to data limitations. Exactly what is mediated in the association is, however, difficult to assess. Importantly, however, the reform did keep the school system intact and did not imply any major changes in learning outcomes or curricula. The instructions to the school districts were to distribute the pre-reform compulsory school curricula in seven years instead of six. The major consequence with the reform was one further year of compulsory schooling.

If more education is causally linked to a reduced risk in premature mortality, this is an argument in favor of longer education for the individual. Our findings give a first indication that compulsory schooling reduced mortality risk. Future research should try to identify the mechanisms in this relationship. It would also be of great interest to separate between death causes to fully understand how education improves health outcomes.
